# Risk Minimization in Scale-Up of Biomass and Waste Carbon Upgrading Processes

**DOI:** 10.1021/acssuschemeng.3c06231

**Published:** 2023-12-30

**Authors:** Jacob H. Miller, Claire T. Nimlos, Yudong Li, Andrew C. Young, Peter N. Ciesielski, Liz M. Chapman, Thomas D. Foust, Calvin Mukarakate

**Affiliations:** † Catalytic Carbon Transformation and Scale-Up Center, National Renewable Energy Laboratory, Golden, Colorado 80401, United States; ‡ Renewable Resources and Enabling Sciences Center, National Renewable Energy Laboratory, Golden, Colorado 80401, United States

**Keywords:** Scale-up, sustainable engineering, catalysis, biochemical upgrading, profit, information

## Abstract

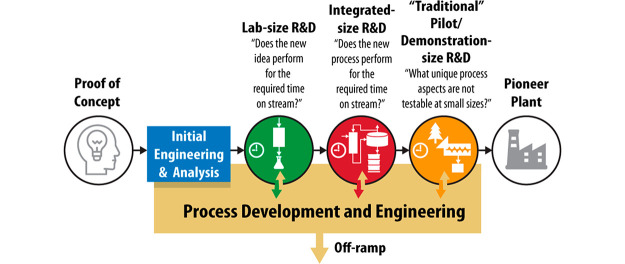

Improving the odds and pace of successful biomass and waste carbon utilization technology scale-up is crucial to decarbonizing key industries such as aviation and materials within timelines required to meet global climate goals. In this perspective, we review deficiencies commonly encountered during scale-up to show that many nascent technology developers place too much focus on simply demonstrating that technologies work in progressively larger units (“profit”) without expending enough up-front research effort to identify and derisk roadblocks to commercialization (collecting “information”) to inform the design of these units. We combine this conclusion with economic and timeline data collected from technology scale-up and piloting operations at the National Renewable Energy Laboratory (NREL) to motivate a more scientific, risk-minimized approach to biomass and waste carbon upgrading scale-up. Our proposed approach emphasizes maximizing information collection in the smallest, most agile, and least expensive experimental setups possible, emulating the mentality embraced by R&D across the petrochemical industry. Key points are supported by examples of successful and unsuccessful scale-up efforts undertaken at NREL and elsewhere. We close by showing that the U.S. national laboratory system is uniquely well equipped to serve as a hub to facilitate effective scale-up of promising biomass and waste carbon upgrading technologies.

## Abstract 2

We provide a framework to minimize risk and shorten timelines of scaling up biomass and waste carbon utilization technologies.

## Introduction

Existential issues stemming from climate change^1^ and ecological accumulation of waste materials^2−4^ compel humanity to develop and implement technologies which replace energy sources and materials derived from petroleum, coal, natural gas, and other fossil sources with low-carbon-intensity alternatives as rapidly as possible. A significant subset of these developing technologies, with applications spanning from transportation fuels^5−9^ to critical materials,^10−12^ relies on upgrading biomass or waste carbon via thermo- or biochemical processing.

The robust processes that upgrade petroleum into critical products have been developed incrementally over the past century, largely using a deliberate, empirical methodology based on proving the performance of processes in a series of units of increasing size (“sizing up”). Merrow et al.^13^ explain this process in a report investigating common scale-up stumbling blocks in the energy and chemicals industry. Successful as this R&D model was, even the petrochemical industry itself is shifting away from it because it often requires ten or more years from lab-scale proof of concept to successful construction of a pioneer plant.^13^ This timeline is also far too slow to accomplish the rapid decarbonization of the entire energy and chemical sector needed in the coming decades to avert calamitous environmental and societal outcomes. The International Energy Agency’s (IEA) sustainable development scenario sets ambitious targets for transition timelines, calling for global CO_2_ emissions to peak at ca. 35 GT/year in the early 2020s and decline to below 10 GT/year in 2050.^14^ A deliberate, empirical approach is also incompatible with the timelines favored by typical start-up investment strategies.

Conversely, many attempts to scale up promising biomass and waste carbon upgrading technologies on accelerated timelines have resulted in the construction of pilot plants that perform less successfully than originally estimated and/or pioneer plants that have higher than anticipated costs, leading to technology abandonment. The IEA estimates that 81% of clean energy start-ups that received seed funding in 2010 failed or sold themselves cheaply, illustrating the prevalence of scale-up failure.^15^ Inexperienced technology developers endeavoring to achieve the energy and materials transition as rapidly and cost-efficiently as possible thus find themselves “between a rock and a hard place”, needing to simultaneously (i) minimize scale-up costs and timelines and (ii) achieve a greater degree of rigor in scale-up approach than has been widely accomplished in the past. Luckily, accomplishing both of these goals is possible. For example, technology developers in the petrochemical industry have recently built a commercial hydrotreating reactor directly from data obtained in lab-sized equipment (scale factor: 3 × 10^6^).^16^

These accomplishments do not happen by chance—increasing the likelihood of accelerated scale-up success requires an innovative strategy steeped in science and engineering fundamentals tailored to confront the unique challenges presented by “new” (relative to petroleum and other fossil sources) biomass and waste carbon feedstocks. We present in this Perspective a framework for technology developers to accomplish thorough, rapid, and cost-effective scale-up of biomass and waste carbon upgrading. In this context, we use an inclusive definition of “technology developer” to refer to anyone (engineers, scientists, technicians, analysts, etc.) involved in a scale-up enterprise. This framework acknowledges the necessity of satisfying two competing obligations that technology developers face:

(i) Demonstrate to external funders, some of whom lack extensive technical backgrounds, that a process is scalable and economically viable, generating sustained external interest and funding to enable the eventual design and construction of a profitable pioneer plant.
(ii) Collect information that enables rigorous evaluation and resolution of process risks, allowing for a pioneer plant to successfully operate as intended.

Many of the findings generated for obligation (ii) will perniciously appear to funders without extensive technical backgrounds to be failures because they increase the estimated process cost. For example, carefully studying catalyst deactivation may increase reactor size and cost estimates or necessitate an additional feed pretreatment step with added associated costs.^17^ This work will demonstrate the necessity of performing information-oriented studies, especially in early scale-up stages (obligation ii) and how these studies are essential to make a process economically viable in the long run (obligation i).

This Perspective explains an alternative to the traditional framework of gradually “sizing up” biomass and waste carbon upgrading processes. We first summarize the reasons why scale-up efforts fail and tie them to a key duality: profit versus information. Then, we show the economic and timeline-based motivation for performing scale-up-oriented research and development in the smallest process units that can be feasibly used. These two points motivate our approach, which stresses up-front evaluation and mitigation of process risks, especially those of durability with time on stream and integration, in the smallest feasible equipment. Finally, we offer an outlook on desirable features and capabilities for flexible piloting facilities and point out the utility of having such capabilities available in publicly funded institutions, such as the US national laboratory system. We envision that this framework can inform decisions made by players across the biomass and waste carbon utilization space, from developers scaling up promising technologies to external funders.

## Sources of Failure in Scale-Up of Biomass and Waste Carbon Conversion Processes

**Scheme 1:**
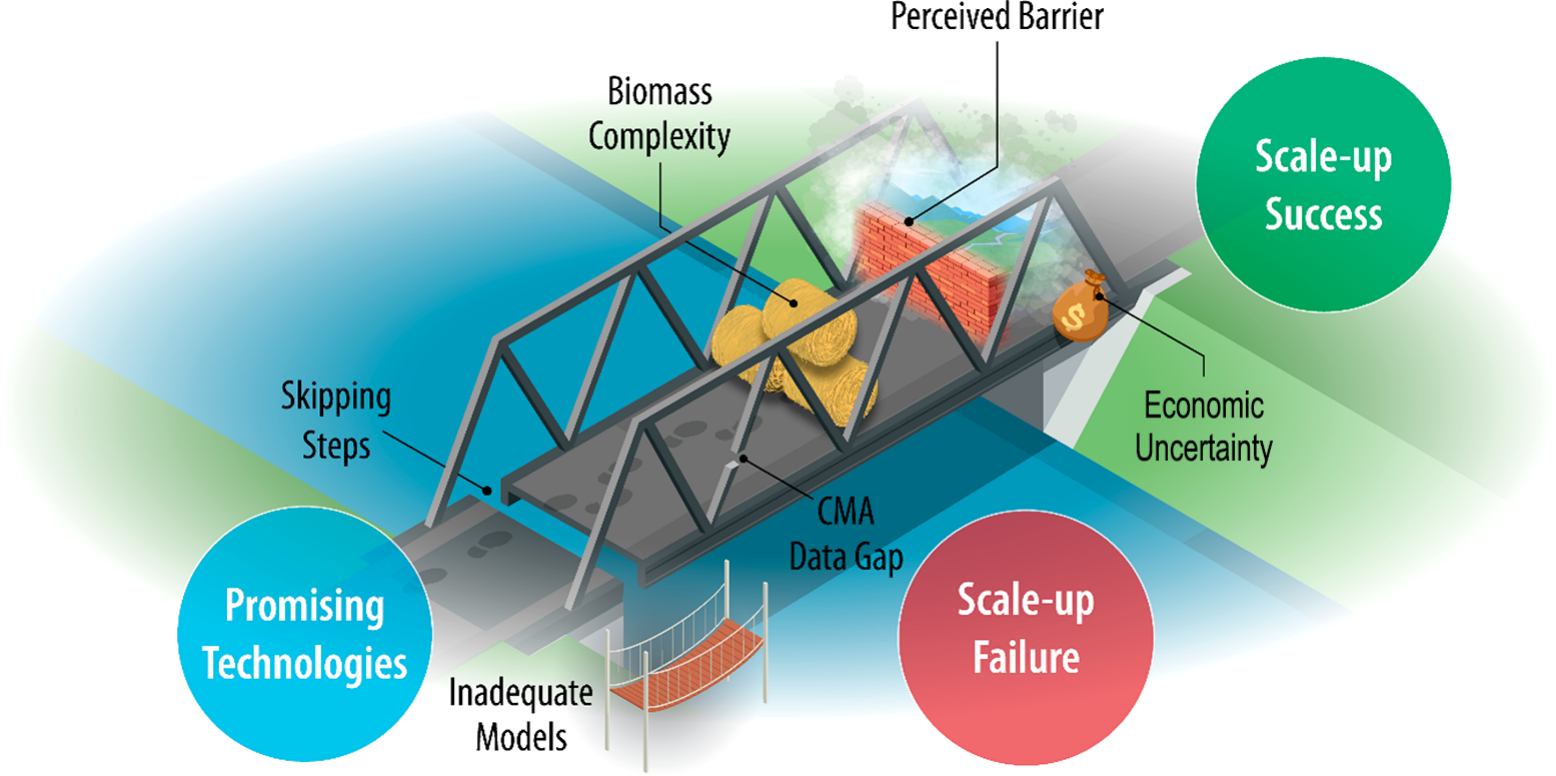
Illustration of Common Modes of Scale-Up Failure

We define failure in scale-up as a range of scenarios in which a promising process is initially deemed to be technically and economically feasible but is abandoned after further research and development, often after significant investments in time, money, and supplies. Merrow et al.^13^ attribute many scale-up failures to cost growth (increased cost of capital equipment or operating costs relative to initial estimations), process uncertainty (lack of knowledge of how to execute an envisioned unit operation), and project uncertainty (changes in scope defined by project leadership independent of technical roadblocks). The IEA adds that unrealistic timelines set by funders, who are accustomed to the faster development pace of other industries such as medical devices, finance, or information technology, and company leaders, eager to promise these timelines to secure funding, also contribute to failures.^15^ We list common biomass- and waste carbon-specific scale-up failures that underpin the failure modes mentioned below and in Scheme 1. We highlight that the likelihood of failure increases when a scale-up team does not include members with relevant expertise to assess each failure mode.

1. *“Skipping steps”.* When a large, capital-intensive process fails to operate as designed, the deficiency is often attributed to failure to either properly test a problematic unit operation or several integrated unit operations at a smaller size, a “step” which was “skipped”. These errors cause fatal process flaws to be overlooked, resulting in construction of expensive pilot or demonstration plants which do not work. Conversely, these errors can also cause fixable flaws to be overlooked until solutions to the flaws are no longer feasible due to extended timelines and high costs of implementing them in large equipment. One especially costly error is attempting to mitigate a process flaw only in “the next” (larger) unit—this approach carries substantial risk that the new fix will also fail at an even higher cost. Regrettably, the most obvious signs of skipping steps only appear after steps have already been skipped.
2. *Critical material attribute data gap*. Basic thermodynamic, rheological, materials compatibility, and other properties of petroleum-derived feedstocks, accumulated via more than a century of both systematic study and trial and error, are broadly available in chemical process development reference resources and software packages. This information guides engineers’ selection of acceptable process conditions and materials of construction. The comparatively wide breadth of biomass and waste carbon feedstock sources and often high variability in properties even within one “feedstock” (e.g., corn stover^18^) have both (i) prevented the same systematic tabulation of biomass and waste feedstock properties and (ii) heightened the uncertainties around estimated properties, dramatically increasing the risk of process failure due to poor understanding of material attributes. One particularly common failure mode stemming from this gap is buildup of initially undetected trace contaminants unique to biomass in process equipment over time.
3. *Relative complexity of biomass processing compared to petroleum*. Technology developers with extensive understanding of only petroleum processing capabilities may overlook issues unique to biomass and waste feedstocks. These issues, especially solids handling, as petroleum is generally a liquid while biomass and waste are usually solids, often must be tackled with skillsets not commonly applied in fossil resource upgrading.
4. *Lack or inappropriate application of unit operation models*. Simulation tools such as reactor models are valuable assets for process scaling, troubleshooting, and optimization. However, computational methods, assumptions, and parametrization schemes developed for petroleum engineering typically do not transfer readily to biomass and waste carbon conversion. Neglecting to adequately collect the necessary data to appropriately model new unit operations can lead to an incomplete understanding of their behavior and how it changes with scale. Thus, inappropriate application of models ported from petroleum processes can be dangerously misleading.
5. *Perceived activation barrier.* Often, “scaling up” is conflated with “sizing up”. Technology developers often neglect to utilize lab-size equipment to explore critical risks. Instead, undo emphasis is placed on efforts to build larger, more expensive “process development units” to explore the same risks, but collection of necessary information in these large units is often prohibitively costly.
6. *Economic and policy uncertainty*. Petroleum and CO_2_ emissions avoidance incentive prices are quite volatile, as the prices of oil and California’s Low Carbon Fuel Standard credits have varied by roughly $100/bbl (ca. $20–$120/bbl) and $160/MT (ca. $60–$220/MT), respectively, throughout the last four years.^19,20^ This makes the comparative future economic viability of competing biomass and waste carbon feedstocks quite difficult to assess, especially for potential investors aiming for short- and long-term profits.

**Scheme 2:**
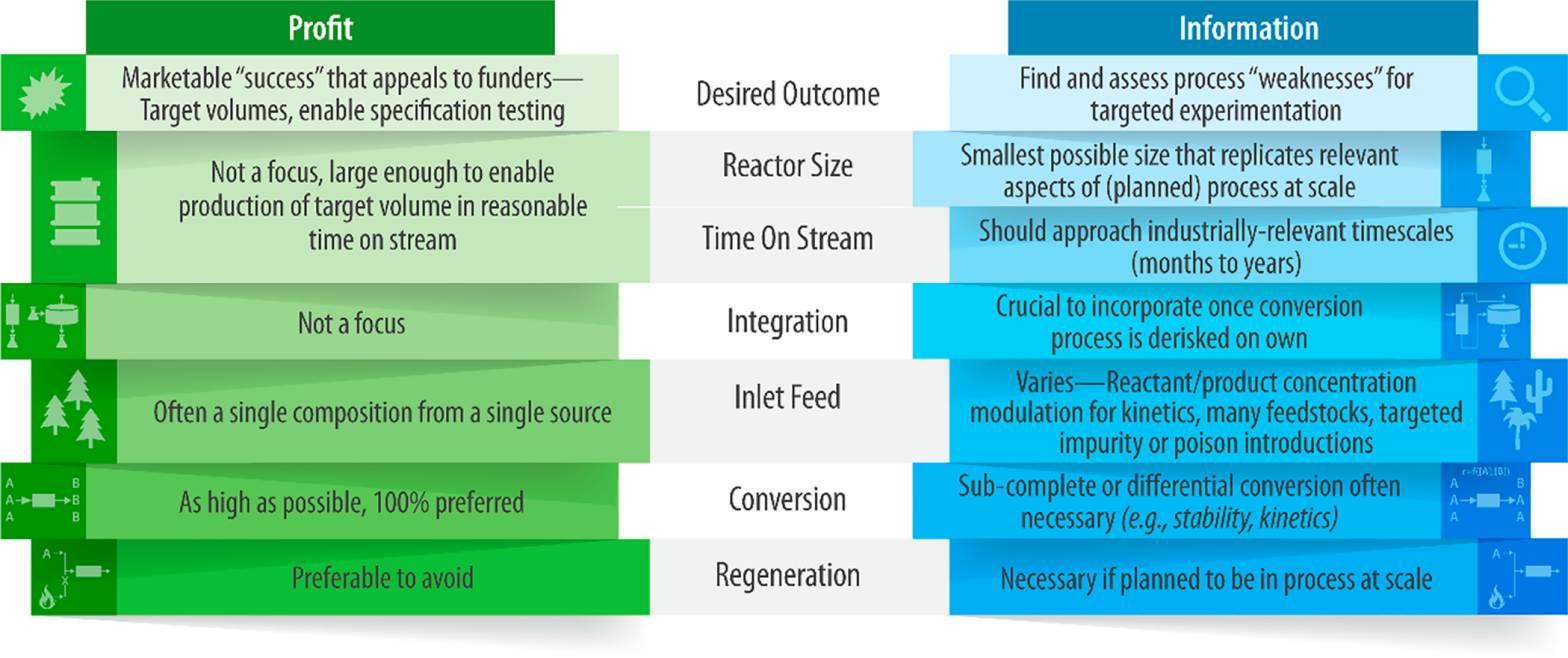
Profit vs Information in R&D Reactors

Each of these failure modes can be understood through a single duality: profit versus information. Petrochemical industry research and development has long embraced the mentality that upgrading processes can be run for profit or information—usually not both.^21^ Successful scale-up is enabled by efficiently collecting information to inform design of commercial plants, often in lab experiments, and petrochemical industry technology developers have become adept at this. The culmination of successful collection and collation of information in scale-up is a properly functioning, profit-generating refinery or chemical plant, although information collected from already functioning plants can spur further increases in throughput and efficiency. We emphasize that laboratory-sized experiments are also sometimes run for profit. Technology developers are often compelled to run small reactors and other units in ways that demonstrate “success” to attract support from prospective funders (research profits); such activities often do not yield new information about the chemistry or physics of the process being developed. Scheme 2 outlines the differences in approach between profit- and information-motivated lab research. Early stage R&D motivated by profit at the expense of information strives to demonstrate marketable “successes”, while information-motivated R&D seeks out process “weaknesses” for further study and remediation. Most major decisions made by technology developers during scale-up are related to at least one of the factors in Scheme 2, and the motivation behind these decisions can be understood through the lens of the profit-information duality.

Inexperienced technology developers emphasizing the pursuit of profit in place of information can fall victim to all six failure sources introduced earlier in this section. Specifically, these technology developers can do the following:

- Place too much trust in the results of profit-motivated experiments run under conditions not representative of the eventual process, resulting in “skipped steps”.
- Ignore the uncertainties caused by the (i) critical material attribute data gap or (ii) relative complexity of biomass processing compared to petroleum.
- Place undue reliance on models for petrochemical upgrading equipment due to the dearth of appropriate models to describe biomass and waste carbon upgrading unit operations.
- Overemphasize the perceived activation barrier that scale-up-oriented experiments must be performed in “sized-up” process development units. As a result, they can underestimate the importance of collecting crucial data in small, inexpensive process units which can be deployed quickly (see next section), instead wasting time, effort, and resources building larger units designed based on inadequate technical information.
- Have the economic prospects of their pathways scuttled by external economic and policy factors. Rigorous forecasting and techno-economic analysis, however, can blunt this risk. An in-depth review of techno-economic analysis is not included as part of this Perspective.

The list of failure modes in this section is likely incomplete—new roadblocks will inevitably arise as more novel technologies are scaled up. Technology developers should look out for these “unknown unknowns”,^22^ but some will always be unanticipated. The approach detailed below allows for technology developers to discover roadblocks in the least expensive way possible, allowing them to build subject matter expertise without the risk of excessive sunk R&D costs.

## Economic and Timeline-Based Motivation for Minimizing Scale-Up Unit Size

**Figure 1:**
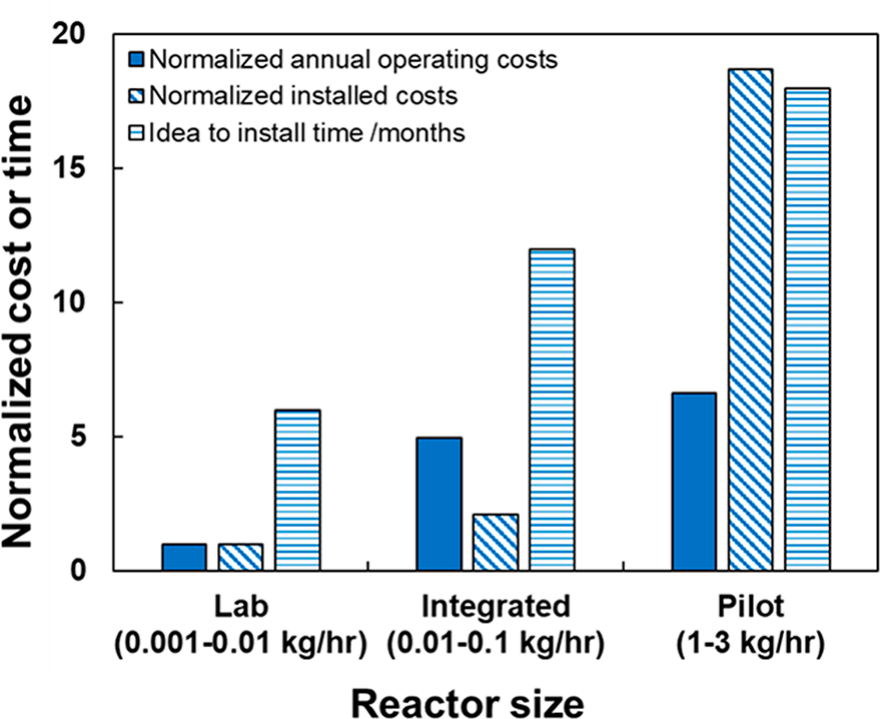
Operating and installed costs (normalized to a lab-size unit) and construction timelines of laboratory, integrated, and pilot-size conversion units at the National Renewable Energy Laboratory.

The desire to minimize costs and timelines of biomass and waste carbon upgrading scale-up motivates us to re-examine the units in which processes are developed. In this section, we review the timelines and expenses associated with operating scale-up units of different sizes. Figure 1 illustrates the operating expenses, capital costs, and construction timelines of three thermochemical biomass upgrading units at the National Renewable Energy Laboratory (NREL). Operating and capital costs are normalized to those of a lab-sized reactor. These numbers vary on a case-by-case basis but are broadly representative of these three unit sizes. The figure shows that integrated and pilot units cost significantly more to operate (5 and 7 times, respectively) and build (2 and 19 times, respectively) than lab-size units. Perhaps even more crucially, they take substantially longer to design and build (12 and 18 months compared to 6 months). Many factors contribute to these differences, including the fact that larger units need more detailed and complex designs, carry more substantive safety risks, and necessitate more (and often more extensively trained) operators.

The economic rationale for *maximizing* unit size, usually applied to commercial plants, still holds true in R&D units—on a basis of money spent per reactor throughput, lab-sized units cost between 15 and 450 times more to operate and 5–160 times more to build than pilot units. However, viewing processes designed for R&D through this *profit*-focused lens is misguided, as they should be operated to gather *information*. We will show in the next section that many pieces of critical scale-up information can be collected in units of any size. Figure 1 shows that scale-up requires significant monetary investment and lead time, inherent risks that, like any others, are crucial to minimize. Since smaller units can be built much faster and more cheaply than larger ones, the imperative for technology developers to collect data in the smallest units possible is clear.

We note that performing scale-up research in existing units with operating envelopes flexible enough to accommodate novel feedstocks and conditions eliminates or minimizes the capital costs and construction timelines discussed in this section. Utilizing these types of adaptable equipment is appealing, but we urge technology developers to perform necessary engineering studies that determine whether the equipment addresses the relevant risks associated with research aims. For example, technology developers in the chemical industry have shown that fluid flow and heat and mass transport characteristics of lab-sized fluidized bed units do not consistently match characteristics of commercial fluidized bed reactors.^21^ Instead, the combination of a lab-size fixed-bed reactor to determine chemical kinetics and one or both of computational fluid dynamics modeling and data from cold flow or tracer experiments is best suited to address the risks associated with scaling up a commercial-scale fluidized-bed reactor. We provide an overview of our scale-up framework to address technological risks in the next section.

## A Risk-Minimization-Focused Approach to Scale-Up of Biomass and Waste Carbon Upgrading

**Scheme 3:**
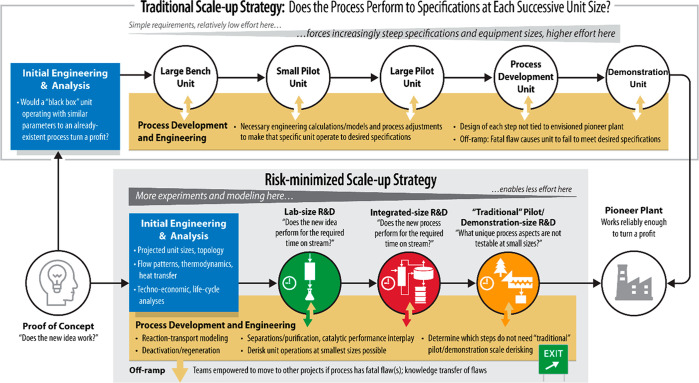
Advantages of a Risk-Minimized, Information-Based Approach to Scale-Up (Bottom Workflow) Compared to the Traditional, Empirical Scale-up Strategy, “Sizing up” (Top Workflow)

In this section, we introduce a time- and risk-minimized process for scientifically approaching scale-up of novel biomass and waste carbon upgrading technologies. This framework, and the contrast between it and traditional scale-up strategies, is shown in Scheme 3. The progression of steps discussed here bears a superficial resemblance to traditional scale-up approaches, as it does invoke a gradual increase in the sizes of process units. We stress, however, two factors that distinguish it: (i) the emphasis on front-end engineering design (FEED), techno-economic, and life-cycle analysis studies, which in turn inform targeted experiments and model validation at each unit size, and (ii) the imperative to probe risks in the smallest process units possible.

### 1 Proof of Concept

All new processes stem from at least one novel idea that has never been implemented at scale before. At the proof-of-concept stage, answering only one question is necessary: *“Does the new idea work?”* Quite often, this is a new reaction or catalyst (biological, thermochemical, etc.) developed in lab experiments without scale-up necessarily in mind. In this case, the lab-scale instrumentation used to prove the idea may be irrelevant to the instrumentation envisioned for commercial implementation (e.g., batch systems instead of flow systems); this is acceptable. As long as the new idea achieves promising metrics (high selectivity or conversion, decreased energy demands, etc.), it is worthy of further examination.

Alternatively, some new conversion concepts may not involve any specific new unit operation, being based instead on using already-proven unit operations in new contexts (e.g., adding a recycle loop to a unit operation already used commercially). The Phillips 66 Rodeo Refinery, for example, will repurpose hydrocrackers and existing downstream operations already proven in crude oil intermediate processing for upgrading pretreated renewable feedstocks to low carbon intensity fuels.^23^ In many such cases, lab-scale proof-of-concept experimentation may not even be needed; simple mass and energy balances or other calculations alone may be sufficient to justify commencing derisking work in lab- or pilot-sized units.

### 2 Initial Engineering and Analysis Study

Scale-up research teams must perform front-end techno-economic analysis, life-cycle analysis, and engineering studies before assuming the risk of commencing scale-up oriented experimentation. These studies must answer two intertwined questions:

(i) Based on the available performance data, what would the design of a pioneer plant look like?
(ii) Would such a pioneer plant be profitable and meet desired sustainability metrics?

Profitability is difficult to define in the abstract, but we encourage technology developers to target the metric sought out by their funders (e.g., a specified internal rate of return) and perform sensitivity analyses around key cost, performance, and economic parameters (e.g., historical commodity prices). The process should be considered potentially profitable if the metric can be reached in 80% of the modeled scenarios.

If the answer to (ii) is “yes”, the engineering team must use the proposed plant design to motivate subsequent risk-minimized scale-up experimentation to adequately address all perceived risks. Specifically, the team should pursue a “scaling down” approach: First, the pioneer plant design concept should be developed enough to project specifications and process conditions for each unit operation (sizes and flow regimes, predicted uptime, methods of regeneration, and required materials of construction), although many of these will be uncertain at this stage. Quite often, this design can be readily formulated in a process simulator such as ASPEN Plus. We note, however, that many process simulators do not have adequate design parameters and flexibility for some units, especially nonstandard reactors or those that exhibit complex fluid mechanics. These are best simulated using computational fluid dynamics (CFD) or other first-principles-derived modeling techniques, the output of which can be incorporated into process simulations to improve the accuracy of unit operation blocks. Technology developers should identify each uncertainty, determine what information would resolve it, and design experiments to collect this information. Many uncertainties, such as lack of thermodynamic data for biomass feedstocks, can stem from the critical material attribute data gap discussed above. Technology developers should make the best guesses for feedstock properties that they can, such as estimating heats of hydrodeoxygenation via feedstock oxygen content or performing targeted calorimetry experiments while incorporating reasonable error estimates. These estimates can be revised as the scale-up continues.

The team must then determine the minimum equipment sizes required and the conditions (flow regimes, pressure, temperature ranges, etc.) at which these experiments can be run, often by matching key dimensionless parameters between envisioned experimental and commercial units. In process engineering parlance, the type of work suitable for this stage would fall into stages 1 and 2 of front-end loading (FEL).^24^ Technology developers should specifically plan at this stage for the implications of endotherms and exotherms stemming from reactions, including maximum reactor diameters to prevent thermal runaway, heat exchange necessities, and thermal stress on catalysts. We identify information best collected for specific equipment sizes in the next three sections.

Sustained engineering and analysis efforts are crucial throughout the scale-up process. The pairs of white and tan arrows in the bottom workflow of Scheme 3 emphasize the importance of frequent information exchange between team members working on experiments, process engineering, techno-economic analysis, and life-cycle analysis. This collaboration will work best in an environment that encourages flexibility: Some experiments to investigate risks such as those outlined in the following sections will reveal a flaw that renders a process infeasible for performance, economic, or life-cycle reasons (e.g., projected process throughput becomes too low, a prohibitively expensive feedstock cleanup step becomes necessary, or the process energy demands become excessively high). Unfortunately, when continuation of a team’s funding is directly tied to the “success” of a specific project, technology developers are disincentivized from identifying these unresolvable roadblocks. If the issues are truly unresolvable, they will cause scale-up to fail after more research, funding, and time has been needlessly devoted to a project. Research teams should thus be encouraged to objectively point out fatal flaws in a process during scale-up without funding for their positions at stake. We stress that the “unfinished” research performed here can still be useful, as best practices and feedstock characteristics generated by one project are often translatable to derisking future projects, and the need to circumvent sources of failure can motivate basic research.

### 3 Lab-Size R&D

The previous section showed that the most cost- and timeline-efficient scale-up-oriented experiments are in lab-sized units with throughputs of 10s–100s of grams per day. Furthermore, advances in multiscale modeling and simulation tools designed specifically for bioenergy processes further enable data collected in lab-sized units to inform robust predictions of process performance across length and time scales.^25^ We emphasize the essential role of lab-size R&D in scale-up in this section; the plethora of information which can be collected efficiently in units of this size shows that maximizing lab-scale R&D utilization and performing accompanying modeling will minimize design uncertainty and experimental necessities for larger units, minimizing overall process development cost and timeline.

One crucial question which can be answered in units of this size is “Does the new idea work *for the required time on stream*?” Uncertainty in the durability of chemical conversion steps, especially catalyst deactivation, enzyme inhibition, and cell culture contamination, is particularly well-suited to be investigated in lab-sized equipment. The molecular-scale processes which most often cause process yields to decrease over time (e.g., carbon deposition, poisoning, nanoparticle sintering, catalyst phase change, and material degradation) can be observed with equal validity at any process size unless accumulation of contaminants is caused by a recycle stream (see next section) as long as heat, mass, and energy flow regimes in all units are equivalent. The small size and comparatively low costs of lab-sized units also enable technology developers to compare the durability of multiple conversion process options at this stage before an informed down-selection.

Deactivation must be monitored over time scales relevant to industrial use; for new processes this is 4000–8000 h at the very least.^26^ Monitoring deactivation in lab-sized units for roughly half of the envisioned lifetime of the commercial process is usually adequate to give technology developers confidence in its viability. We specifically recommend monitoring the deactivation of chemical reaction processes at partial reactant conversion, as it is impossible to discern how much, if any, of a catalyst loading has deactivated over time while running at full conversion. If running at full conversion is unavoidable, we urge technology developers to not rely on reaching a time-on-stream metric alone but instead upon criteria based on total output per catalyst mass (e.g., mass of product per catalyst mass in thermal catalysis or enzyme loading in biochemical conversion, where 10^3^–10^4^ is desirable). As technology developers gain a firmer understanding of process durability, we encourage frequent exchanges of information with techno-economic analysts to ensure that projected unit lifetimes are economically feasible. These collaborations can also motivate studies of regeneration cycles (e.g., burning coke from heterogeneous catalysts) to ensure that processes remain durable through regenerations and inform unit design decisions such as identifying whether a thermochemical catalytic reaction is best suited to take place in a fixed-bed, swing-bed, or recirculating riser-type reactor.^27^

A prevalent cause of deactivation during biomass upgrading is catalyst or membrane poisoning from biogenic impurities.^17,28^ We recommend that feeds to lab-sized equipment are as similar as possible to envisioned pioneer plant feedstocks, such that technology developers can identify as quickly as possible which impurities are harmful to a conversion process. Thus, technology developers must introduce real feedstocks with as many variations in source, purity, and composition as possible when testing process durability in lab-sized equipment. Additionally, trace particulates or other components of real feedstocks are often responsible for equipment plugging, another important phenomenon that can be investigated (and mitigated) in lab-sized units. Degradation resistance of process equipment such as reaction and holding vessels is also suitable to observe in lab-sized experimental units, as material corrosion behavior does not vary with unit size.^29,30^ Results of these experiments can inform the project team of the necessity to use certain materials in specific process units, specifying pioneering plant capital cost estimations.

Process economic viability is strongly correlated to unit sizing; this is crucial to determine early in development. Kinetic measurements of reactive processes determine commercial unit sizing by showing the impact of reactant, product, and impurity concentrations on conversion rates; therefore, these studies are best suited for lab-sized reactors. While it is impossible to operate lab-sized processes with attributes identical to those planned for larger units, this is not always necessary or even preferred at this stage. Instead, kinetic parameters obtained in lab-sized units must be measured under conditions devoid of heat and mass nonuniformities such as diffusion limitations within catalyst pellets or bulk transport phenomena within the reactor. Kinetic parameters collected from these experiments can be incorporated in multiscale models to inform design of integrated, pilot/demonstration, or commercial equipment which reintroduce heat and mass transfer limitations. In many cases, lab-sized reactor configurations such as well-mixed continuous stirred tank reactors or differential plug-flow reactors can be operated essentially free of heat and mass transfer limitations. Although these lab-sized units may look quite different from the intended configuration on larger scales, the information obtained is likely far more useful than that obtained from simply building a smaller version of the envisioned commercial or pilot unit. The acquisition of conversion and deactivation kinetics at this stage provides an important opportunity for further development and parametrization of unit operation and process models that propagate conversion, heat and mass transfer, and deactivation kinetics to larger length and time scales,^31^ further specifying commercial process viability projections and informing the design of larger equipment.

Similarly, examining the effects of the additional phases incorporated in catalyst extrudates (binders, plasticizers, etc.) on reactivity is essential at this stage.^32,33^ However, the dimensions of lab-sized packed-bed reactors often induce prohibitively large heat and mass transfer boundary layers around commercial catalyst extrudates,^34,35^ making kinetic data collected over these materials in lab-sized packed-bed reactors uninformative unless the transport limitations are modeled explicitly and separately from the kinetics using mesoscale simulation methods.^36^ We recommend that technology developers crush extrudates to submillimeter particles of appropriate size for lab reactors to avert mass and heat transfer limitations and collect data over these particles. Reaction rate data collected in this way can be combined with porosity characterization of extrudates to predict their heat and mass transfer limitations,.^37,38^ with the help of CFD simulations to accurately account for hydrodynamics.^39^ Alternatively, engineering-form extrudate performance can also be tested in Berty reactors or other similar lab-sized continuous stirred tank reactors with plug flow hydrodynamics that eliminate external heat and mass transport limitations around extrudates.^40,41^

Catalyst particle wetting is one process feature that technology developers should attempt to render identical between lab-size experiments and envisioned pilot plant operations. Entirely coating catalyst particles in a film of liquid (wetting) in three-phase systems such as trickle-bed hydrotreaters is difficult but crucial to achieve in all equipment sizes. Geometry constraints, especially wall effects, makes achieving total wetting in lab-sized units difficult, but the analysis demonstrated by Mederos et al.^35^ can allow researchers to design fully wetted lab-sized reactors. Meanwhile, industrial equipment like liquid distributors can be deployed to help achieve wetting in larger units.^42^ We recommend that technology developers ensure that lab-sized reactors achieve equivalent wetting to the expected industrial conditions to inform analysis and modeling.

### 4 Integrated-Size R&D

Some scale-up related questions cannot be practically answered in lab-sized equipment. Most prominently, issues related to integration of sequential process units such as testing for accumulation of contaminants in recycle loops require construction of units that operate on at least a scale of kilograms per day; technology developers must utilize integrated units to collect this information in spite of the increased associated costs and timelines compared to lab-size equipment. Information derived from operating processes at sizes sufficient for process integration should answer questions about how novel unit operations work in the context of the broader process: “Does the new *process* work for the required time on stream?” If a new process does not contain any novel unit operations and the novelty instead stems from a new integration of unit operations, targeted scale-up studies can start with integrated equipment.

One important issue to address using integrated-sized equipment, if not through analysis of individual processes at the lab scale, is the interplay between the performance of different process units. This question is applicable when considering the interaction between separation and catalysis. For example, in hybrid fermentation/thermocatalytic conversion processes, fuel or material precursors generated via fermentation are subsequently fed into catalytic reactors. These precursors exist in an aqueous broth; the water, cell material, and metal salt nutrients in this broth can all potentially harm heterogeneous catalysts and can be separated from the target product via methods such as membranes or hybrid extraction–distillation–*in situ* product recovery^9^ to make extremely pure target molecule streams. However, such separations can be costly and are sometimes unnecessary. Technology developers should determine whether downstream catalytic processes are found to be unharmed by certain concentrations of these contaminants, reducing the requirements for these processes. This can lower the process energy demand, materials demand, and overall costs.

As with the lab-sized units discussed above, integration-sized units should be designed to derisk key features of the eventual pioneer process, and data obtained from these studies should add specificity to engineering, techno-economic, and life cycle greenhouse gas emissions models. Process units at this size will be of sufficient dimensions to include features similar to their pioneer plant analogues. For example, reactors should be large enough to contain engineering-form catalysts with flow patterns relevant to commercial reactors. At this stage, process feeds should exclusively be biogenic or waste-derived; model compound studies have limited utility.

Integration-sized units are also good settings to conduct “stress tests”, in which temperatures and pressures of specific subprocesses (e.g., reactors or distillation columns) are perturbed to simulate feasible process disturbances, allowing for observation of subunit responses and corresponding adaptations or planning for such contingencies. If technology developers suspect that a specific unit operation is sensitive to disturbances, a lab-size version of it alone can also be stress tested.

Problems such as catalyst activity/selectivity decreases, mechanical failure, or equipment fouling can decrease yields of integrated processes enough to force a shut down or throughput reduction. Integration-sized units are optimal for determining the necessary time at which an integrated process must be taken offline to fix these problems, known as the cycle length, and developing design solutions to prolong it. The data gathered from integrated process operation should be used by technology developers to redesign equipment to enable prolonging of cycle lengths (i.e., using parallel or fouling-resistant heat exchangers) if extra equipment capital costs are feasible. Operating these integrated-sized units for long periods of time (hundreds of days) allows for technology developers to determine strategies to increase cycle length at the smallest feasible process size.

Waste mitigation costs can be significant; processes utilizing biomass feedstocks with high water content or high oxygen content if deoxygenation is performed will inevitably produce large quantities of wastewater. The implications of this should be directly examined using integrated or smaller-size equipment. We recommend that technology developers measure pH, chemical oxygen demand, biological oxygen demand, total suspended solids, ammonia concentration, total Kjeldahl nitrogen, total phosphorus, peak flows, and total flows of wastewater streams from integration-sized equipment, as these quantities are used for cost and sizing of wastewater treatment systems. Methodologies for handling waste solids, especially hazardous wastes, on a commercial scale are less widely understood, but we recommend that technology developers be cognizant of the solid wastes they produce and consult with waste management companies on cost-effective mitigation strategies.

Modeling based on data obtained from integrated-size units can be instrumental in specifying the design of larger units and even point out fatal flaws in process design. For example, a recent collaborative study between NREL and Oak Ridge National Laboratory examined the thermal effects of oxidatively regenerating a fixed bed containing coke-covered Pt/TiO_2_ catalyst used for catalytic fast pyrolysis from data obtained in an integrated biomass pyrolyzer and fixed-bed reactor with a capacity of ca. 3 kg/day.^27^ Researchers specifically sought to evaluate the risk of overheating the catalyst during coke combustion, which would render it permanently inactive. Researchers performed parameter estimation based on integrated system data and modeling of heat transfer during coke combustion to find that, although the temperatures observed in the integrated-scale reactor were not severe enough to irreversibly damage the catalyst, heat transfer limitations of a larger (higher-diameter) pilot-size reactor would irreversibly damage the catalyst, even if the pilot reactor had internal cooling tubes installed. This study prevented a future scale-up misstep that could have otherwise been attributable to our fourth mode of failure, lack or inappropriate application of unit operation models, and allowed the project to pivot and utilize a different, more thermally stable catalyst.

Occasions will often arise in which technology developers must run lab- or integrated-size processes for profit instead of information. For example, it is often necessary to generate a sufficient volume of a final product to conduct physical property testing. In other settings, the need for careful study of one unit operation necessitates running upstream processes at full conversion to simply generate enough feed for that study. We encourage technology developers to derive as much information as they can from these types of experiments but to recognize that many necessary features of these experiments are at odds with maximizing information output. One prominent feature difficult to test even in integrated equipment running at ca. 1 kg/day is the handling of solids; this major challenge often must be tackled at the “traditional” pilot scale.

### 5 “Traditional” Pilot/Demonstration-Size R&D

Traditional pilot- or demonstration-sized process units are designed to operate at throughputs higher than those of integrated-size instrumentation. We define this here as upward of 1 kg/h, although the DOE Bioenergy Technology Office typically defines pilot equipment as having a throughput of at least ca. 1 tonne/day (42 kg/h).^43^ We emphasize that the size of pilot (or demonstration) equipment should be defined by the limitations to adequately address risks and not a hard minimum size. Given the elevated costs and timelines associated with the units discussed above, our approach advocates using these process units only to investigate risks that cannot be examined in smaller equipment. One important risk that must be addressed in “traditional” pilot- or demonstration-sized equipment is handling of solids, which often cannot be reliably tested in lab- or integrated-size units. Merrow et al.^13^ identified solids handling 40 years ago as the feature most likely to add risk to novel process scale-up. Today, solids handling is still a major contributor to risk. Research to identify the fundamental causes of issues such as plugging and degradation of solid handling and delivery equipment is needed. Until the underlying causes of these issues are identified, enabling solids handling to be reliably investigated using smaller equipment, solids handling tests must take place in traditional pilot- or demonstration-sized units.

Pilot- or demonstration-sized processes should be integrated, but we stress that not all parts of an integrated process must be tested at the same throughput. If, for example, major risks of a reactor downstream of a solids processing unit have been addressed at smaller sizes (e.g., catalyst deactivation, kinetics, materials compatibility, accumulation of contaminants in recycle loops) and/or with engineering modeling (e.g., thermal management, heat integration), the only new features being introduced by incorporating solids handling are integration of the reactor with the solids handling unit. Risks associated with new features, including reactor plugging and additional catalyst deactivation, can often be observed in laboratory- or integrated-sized reactors. We accordingly advise routing a carefully homogenized slipstream of the solids handling process effluent to a smaller reactor instead of building a larger and more expensive demonstration- or pilot-sized reactor when feasible. Integration of pilot- or demonstration-sized units with smaller units creates a “mixed-size” pilot or demonstration plant. As long as this “mixed-size” plant operates continuously, the entire process can be derisked more quickly and with dramatically lower capital and operating costs than a full-size pilot or demonstration plant. We emphasize the necessity of operating the process continuously. For example, an industrial collaborator worked with technology developers at NREL to scale up a process involving gasifying solids and upgrading the resultant syngas in a bioreactor. The bioreactor was designed to have a lower throughput than the gasifier; therefore, only a fraction of the produced gas needed to be fed to it. When the system was first developed, syngas was captured in pressurized gas cylinders and subsequently fed to the bioreactor. However, the research team realized the importance of testing the system as an integrated unit and built a direct gas transfer line from the gasifier to the bioreactor. This configuration allowed them to observe a contaminant that was previously trapped by the metal of the gas cylinders and design a solution to mitigate it, eliminating the risk posed by the contaminant to the process. This example illustrates the importance of implementing process integration in “mixed-size” equipment.

Experimental process development is sometimes undertaken with pilot- or demonstration-size equipment without collection of data in smaller units. For example, a recent collaboration between NREL and Petrobras investigated coprocessing of pyrolysis oils with vacuum gas oil (VGO) in fluid catalytic crackers (FCCs) using a demonstration-size FCC unit (200 kg h^–1^). The team found that up to 10 vol % bio-oil could be successfully incorporated into the feed stream without hindering unit performance as long as the oil was not aged for more than nine months.^44^ Rigorous techno-economic analysis can also play a major role at this stage as well, as subsequent detailed modeling of data from this study showed that coprocessing of bio-oil was only economical if feedstocks could be obtained at lower prices than pine chips in the United States.^45^ We stress here that such experiments usually are only successful and cost-efficient if (i) the process being developed is extremely similar to an already operating process and (ii) the unit for testing the process is already built or only needs slight modifications. In this case, since the feed was mostly (>90%) VGO and the FCC demonstration unit already existed, the experiments could be performed. This will not be the case for most novel processes, especially when feeds are made up of only biomass, waste, or molecules derived from these sources.

The importance of CFD or other high-fidelity multiphysics simulations of unit operations at this scale should be neither overlooked nor relied upon exclusively. Virtually every modern traditional petrochemical company employs a CFD team to assist in various aspects of process development and deployment, but these by no means preclude the need for experimental counterparts. Still, the increased availability of high-performance computing resources and continual improvements in simulation software have steadily enhanced the ability of CFD codes to accurately portray complex physical processes, such as fluidization of nonspherical particles^46^ and biomass gasification,^47^ with high fidelity. A thoroughly validated simulation of a pilot or demonstration unit is a powerful tool, as it can be used to explore a wide parameter space of operating conditions and even feedstock characteristics, if properly coupled to a mesoscale submodel,^48^ in a rapid and cost-effective manner relative to a corresponding experimental campaign. Such results can be used to estimate safe and optimal operating regimes, identify potential risks, troubleshoot during startup, and even investigate some new configurations.

Technology developers in the petrochemical industry often perform a final step before building a pioneer plant: Running pilot or demonstration processes under the exact conditions to be used in the pioneer plant (full conversion, etc.). Earlier in the scale-up process, this strategy would unequivocally be categorized as placing profit before information. However, if technology developers are confident that they have resolved all feasible risks, running a pilot or demonstration process for “profit” is an effective final check to identify any remaining “unknown unknowns”. In the context of this final checkpoint, running the process for “profit” in pilot or demonstration equipment can also generate information that would be impossible to collect any other way.

### 6 Pioneer Plant Operations

While the goal of all above steps is to maximize information and generate “profit” by collecting enticing results that sustain funding, the exclusive goal of a pioneer plant is to turn a profit by producing the target product(s). Information about a process is also quite often collected in pioneer (and nonpioneer) commercial plants, but this is often relevant only when the process is not functioning properly. Causes for this failure, meanwhile, can often be traced back to risks which could have been resolved or identified as fatal in smaller-sized R&D units. Many nontechnical risks not covered here such as siting, governmental regulations, and supply chain optimization^13^ arise when building and operating pioneer plants, so the goal of all prior R&D steps should be to minimize technical risks at this stage.

**Scheme 4:**
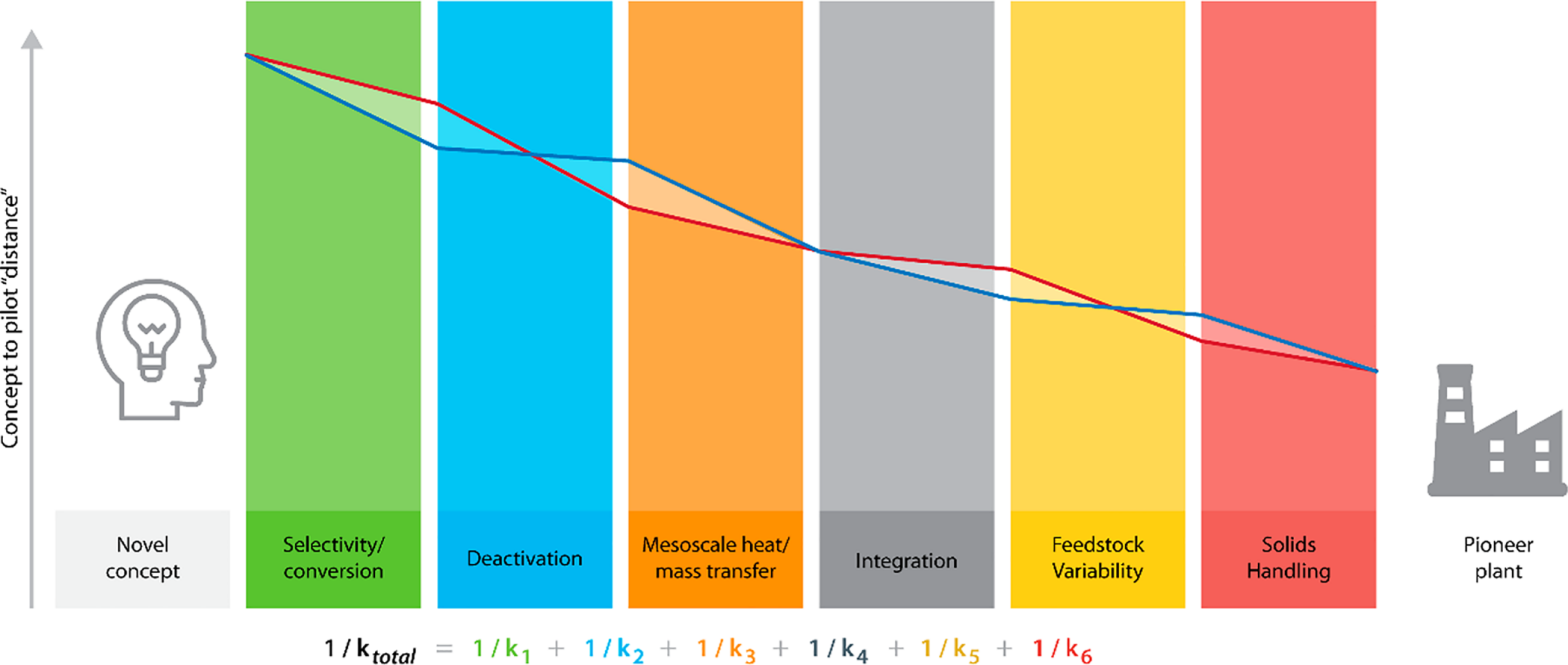
Scale-Up Allegory of Heat/Mass Transfer Resistances in Series for Two Generic Scale-Up Scenarios (Red and Blue Lines)

We conclude our vision for a risk-minimization-focused approach to biomass and waste carbon upgrading scale-up by stressing the importance of building a diverse team featuring members with a variety of relevant areas of expertise and facilitating frequent communication within the team. Assessing the scale-up potential of an entire process can be viewed through the allegorical lens of a common engineering problem: heat or mass transfer through multiple media in series (Scheme 4). Each scale-up risk is a “resistance” (1/*k*_*i*_). As 1/*k*_total_, the total scale-up risk becomes smaller, and the novel process becomes closer to a commercial reality. As shown by the familiar formula in Scheme 4, 1/*k*_total_ becomes smaller as each individual risk *i* (1/*k*_*i*_) is minimized. However, as an individual risk is minimized, decreasing that risk further becomes less and less relevant to reducing the total process risk, as total risk becomes dominated by unaddressed challenges, which still have large values of 1/*k*_*i*_. It is paramount, then, for technology developers considering specific risks not to be “siloed” from one another, unaware of the overall contributions of their work to decreasing the overall barrier to commercialization. Frequent crosstalk between team members with diverse skill sets will allow teams to optimize allocation of their time and resources to lower the total scale-up barrier as rapidly as possible.

## Outlook: Features of a Scale-Up Hub

The scale-up vision outlined in this Perspective is not easy to carry out for even a single process. Even so, humanity must rapidly develop many biomass and waste carbon upgrading technologies simultaneously. These efforts would be aided by a facility at which many users could design processes and investigate risks by using shared equipment of various sizes. Large petrochemical companies possess the equipment and expertise to follow these scale-up steps, but their facilities are not often available to the many smaller start-up companies currently attempting to scale up biomass and waste carbon upgrading technologies. The United States’ national laboratories, such as the National Renewable Energy Laboratory, are well suited to serve these small companies as scale-up hubs, as they contain appropriate equipment (including specialized instruments for biomass and waste carbon feedstock analysis), are without the imperative to turn a profit for themselves, and are staffed with researchers with a diverse array of competencies. Notably, they encompass not only experimentalists but also computational experts, such as the multinational lab Consortium for Computational Physics and Chemistry,^49^ which is dedicated to developing, validating, and applying simulation and machine learning tools specialized for upgrading low-carbon intensity feedstocks. The diversity of equipment and expertise available at national laboratories specifically allow for facile integration of upgrading steps requiring vastly different operating and safety requirements (e.g., biochemical, thermochemical, photochemical, or electrochemical steps) in a single process, a crucial capability for many conversion processes. These features would enable small companies to avoid the costs and timelines associated with building their own R&D equipment; national laboratories also would be well-positioned to facilitate scale-up of future technologies that have yet to be conceptualized.

The envisioned commercialization hub would have resources to help technology developers through all scale-up stages. Technology developers looking to commercialize their processes could first have their initial plant designs and life-cycle and techno-economic analyses vetted and optimized by facility engineers and analysts. Next, the facility would offer various sizes of R&D units with the flexibility to be adapted to various applications, allowing for risk-minimized optimization of all (individual and integrated) process steps. All along the way, scale-up hub experts would be able to draw on centralized knowledge and modeling and machine learning capabilities to steer experimental plans and optimization and integration of process units for each technology. Since most of the design, analysis, and computational capabilities discussed here are digital, many functions of the hub could be performed remotely, decreasing barriers to entry and lowering operational costs. Scale-up hub researchers would meanwhile be empowered to educate the broader community about solutions to common stumbling blocks gained from experience helping technology developers from many backgrounds. All the while, scale-up hub researchers would gradually increase their effectiveness in efficiently commercializing biomass and waste carbon utilization technologies.

The scale-up strategy introduced in this paper, whether executed inside or outside of a commercialization hub, can be utilized to help humanity rapidly implement technologies that utilize biomass and waste carbon sources to satisfy energy, transportation, and materials demands while reining in greenhouse gas emissions.
